# MicroRNA-1281 as a Novel Circulating Biomarker in Patients With Diabetic Retinopathy

**DOI:** 10.3389/fendo.2020.00528

**Published:** 2020-08-04

**Authors:** Marta Greco, Eusebio Chiefari, Francesca Accattato, Domenica M. Corigliano, Biagio Arcidiacono, Maria Mirabelli, Rossella Liguori, Francesco S. Brunetti, Salvatore A. Pullano, Vincenzo Scorcia, Antonino S. Fiorillo, Daniela P. Foti, Antonio Brunetti

**Affiliations:** ^1^Department of Health Sciences, University of Catanzaro “Magna Græcia,” Catanzaro, Italy; ^2^Department of Medical and Surgical Sciences, University of Catanzaro “Magna Græcia,” Catanzaro, Italy

**Keywords:** diabetic retinopathy, type 2 diabetes, miRNA profiling, serum biomarkers, VEGFA, HUVEC endothelial cells, ARPE-19 epithelial cells

## Abstract

**Objective:** Recently, the role of circulating miRNAs as non-invasive biomarkers for the identification and monitoring of diabetes microvascular complications has emerged. Herein, we aimed to: identify circulating miRNAs differentially expressed in patients with and without diabetic retinopathy (DR); examine their predictive value; and understand their pathogenic impact.

**Methods:** Pooled serum samples from randomly selected matched patients with type 2 diabetes, either with or without DR, were used for initial serum miRNA profiling. Validation of the most relevant miRNAs was thereafter conducted by RT-qPCR in an extended sample of patients with DR and matched controls.

**Results:** Following miRNA profiling, 43 miRNAs were significantly up- or down-regulated in patients with DR compared with controls. After individual validation, 5 miRNAs were found significantly overexpressed in patients with DR. One of them, *miR-1281*, was the most up-regulated and appeared to be specifically related to DR. Furthermore, secreted levels of *miR-1281* were increased in high glucose-cultured retinal cells, and there was evidence of a potential link between glucose-induced *miR-1281* up-regulation and DR.

**Conclusion:** Our findings suggest *miR-1281* as a circulating biomarker of DR. Also, they highlight the pathogenic significance of *miR-1281*, providing insights for a new potential target in treating DR.

## Introduction

Type 2 diabetes mellitus is an expanding global health problem, with an estimated prevalence of 8.8% in the general adult population (1), and a prediction of over 500 million affected people in the world by year 2030 (2). Chronic hyperglycemia in both type 1 and type 2 diabetes is associated with the development of long-term microvascular and macrovascular complications that may seriously compromise the patients' quality of life and life expectancy, and can contribute to high healthcare burden and costs (3).

Among diabetes-related microvascular complications (namely retinopathy, neuropathy and nephropathy), diabetic retinopathy (DR) is one of the most devastating and frequent injuries, affecting over 30% of patients with diabetes, and one of the leading causes of vision loss in the working-age population (4, 5). It is frequently the first microvascular complication to appear, and the risk of developing DR is directly related to the duration of diabetes and to the level of metabolic control (6).

In the recent years, a big effort has been made to identify novel biomarkers for both the prediction of diabetes (in particular type 2 diabetes) in individuals at risk of developing the disease, and for preventing the development of diabetes-related long-term complications. Several emerging lines of evidence in this field point to the relevance of circulating microRNAs (miRNAs) as disease markers (7, 8). MiRNAs are an evolutionarily conserved class of short non-coding single-stranded RNAs, which target over one-half of protein-coding transcripts (9, 10). They are involved in fundamental biological processes, such as cell differentiation and proliferation, apoptosis, tissue development, senescence and metabolism (9, 10). Recently, miRNAs have been implicated in the pathophysiology of many human diseases, including type 2 diabetes (10–12), as well as in the inflammatory and endothelial dysfunction that can trigger the development of vascular complications in diabetes (13–16). The application of circulating miRNAs as biomarkers for DR has gained much attention in recent years. In particular, at this level, miRNAs have been implicated in aberrant angiogenic growth of retinal endothelial cells, by adversely affecting the levels of vascular endothelial growth factor A (VEGFA) (17), a secreted mitogen which seems to play a role in DR, by inducing neovascularization and vascular permeability of retinal vessels (4, 18, 19). Research studies in this context have focused on a variety of *in vivo* and *in vitro* retinal models (20–22), as well as on human biological fluids from diabetic retinopathic patients (7, 23–26). However, little consistency emerges across these studies, such that solid conclusions cannot yet be made concerning the relationship between miRNAs and DR.

In the present work, we aimed at investigating miRNA expression patterns in serum samples of highly selected patients with type 2 diabetes, with and without DR. Among the newly identified miRNAs was *miR-1281*, which was found to be strongly associated with DR, and whose pathogenic role in this diabetic complication was also supported by a mechanistic explanation.

## Materials and Methods

A case-control study for the identification of DR-associated plasma miRNA(s) was carried out in two steps. In the first step, an initial screening for serum miRNA profiling was performed. On a second step, we dealt with the individual validation of the most relevant miRNAs. The study was approved by the local ethics committee (Comitato etico regionale, Sezione Area Centro, Catanzaro, Italy, Protocol n. 116, May 14, 2015), and all participants gave written informed consent.

### Enrolled Patients

In the first step of the study, 5 unrelated men with type 2 diabetes and non-proliferative DR, in the absence of other diabetic complications, were enrolled at the Operative Unit of Endocrinology (University “Magna Græcia,” Catanzaro, Italy). A control group of 5 unrelated men with diabetes, but without DR, matched with the case group for clinical and biochemical features [age, BMI, duration of diabetes, blood pressure, glycated hemoglobin (HbA1c), lipid profile], in the absence of other disorders, and receiving the same medical treatment (metformin only), was recruited at the same time period from the same outpatient clinic (Table 1). At this step, only male patients were included to maximize matching between the two groups in consideration of the greater presence and severity of DR in men (27), and the known effects of sex hormones on miRNAs (28, 29). In the second step, 15 further unrelated patients with type 2 diabetes and DR, in the absence of other long-term complications, and 25 matched unrelated patients with type 2 diabetes, but without DR, were included (Table 1). In all participants, type 2 diabetes was diagnosed according to the American Diabetes Association criteria (30), whereas the diagnosis of non-proliferative DR was based on ophthalmological examination of the ocular fundus following dilation of the pupils, as reported before (19). Severity of DR was graded based on the worst eye, according to the International Clinical Diabetic Retinopathy Disease Severity Scale (31). Subjects affected by proliferative DR, or with pan-retinal photocoagulation, were excluded from the study. Diabetic nephropathy was excluded on the basis of an estimated glomerular filtration rate (eGFR) > 60 mL/min/1.73 m^2^, according to the Chronic Kidney Disease Epidemiology Collaboration (CKD-EPI) formula (32), and on the absence of microalbuminuria (33). Neuropathy and macrovascular complications were excluded after appropriate clinical and instrumental examinations.

**Table 1:** Clinical and biochemical baseline features of enrolled patients.

	**First step**		**Second step**	
	**DR (*n* = 5)**	**Controls (*n* = 5)**	**DR (*n* = 20)**	**Controls (*n* = 30)**
Ethnicity	Caucasian	Caucasian	Caucasian	Caucasian
Female (%)	0 (0.0)	0 (0.0)	10 (50.0)	15 (50.0)
Age (yr)	64.6 ± 6.8	65.0 ± 8.0	63.8 ± 6.8	63.8 ± 10.4
Duration of diabetes (yr)	11.4 ± 3.0	11.2 ± 3.7	10.8 ± 3.6	10.9 ± 4.7
BMI (Kg/m^2^)	25.0 ± 2.8	27.6 ± 2.0	27.4 ± 2.4	26.4 ± 2.6
Systolic BP (mmHg)	132 ± 5.7	130 ± 6.1	131.0 ± 8.5	134.5 ± 9.0
Diastolic BP (mmHg)	78 ± 4.5	77 ± 2.7	78.0 ± 5.7	77.2 ± 6.5
Antihypertensive therapy (n)	5 (100.0)	5 (100.0)	15 (75.0)	22 (73.0)
FPG (mg/dL)	136.2 ± 25.2	139.0 ± 13.2	149.2 ± 30.8	150.5 ± 32.3
HbA1c (%)	7.6 ± 0.7	7.4 ± 0.5	7.3 ± 0.9	7.3 ± 0.8
Metformin only (n)	5 (100.0)	5 (100.0)	17 (85.0)	27 (90.0)
Total cholesterol (mg/dL)	168.8 ± 11.1	169.8 ± 14.6	173.2 ± 21.9	168.2 ± 19.5
HDL-C (mg/dL)	49.0 ± 9.7	47.6 ± 9.61	50.0 ± 11.6	46.6 ± 11.8
LDL-C (mg/dL)	95.6 ± 14.2	97.9 ± 12.5	96.9 ± 20.3	98.6 ± 20.1
Triglycerides (mg/dL)	121.2 ± 35.4	121.4 ± 23.8	130.9 ± 41.3^*^	109.9 ± 34.6^*^
Hypolipidemic therapy (%)	5 (100.0)	5 (100.0)	14 (70.0)	24 (80.0)
ESR (mm/h)	19.2 ± 6.8	20.0 ± 8.5	16.1 ± 6.2	17.2 ± 6.6
hsCRP (mg/L)	3.9 ± 0.9	4.0 ± 1.1	4.3 ± 0.9	4.5 ± 1.2
Creatinine (mg/dL)	1.0 ± 0.1	0.9 ± 0.2	0.9 ± 0.2	0.9 ± 0.1
eGFR (mL/min/1.73 m^2^)	79.0 ± 12.6	89 ± 12.6	82.9 ± 12.9	87.0 ± 13.6

Data are mean ± SD, or n (%). Non-parametric Mann-Whitney test was used for distribution comparisons of quantitative variables. The two-tailed Fisher Exact Test was used for proportion comparisons between groups.

* *p < 0.05. BMI, body mass index; BP, blood pressure; FPG, fasting plasma glucose; HDL-C, high-density lipoprotein-cholesterol; LDL-C, low-density lipoprotein-cholesterol. ESR, erythrocyte sedimentation rate; hsCRP, high-sensitivity C-reactive protein; eGFR (CDK-EPI), estimated glomerular filtration rate. To convert mg/dL glucose to mmol/L, multiply by 0.0555. To convert % HbA1c to mmol/mol, multiply by 10.9 and subtract 23.5. To convert mg/dL of total and partial cholesterol to mmol/L, multiply by 0.0259. To convert mg/dL triglycerides to mmol/L, multiply by 0.0113*.

Obesity (BMI ≥ 30 kg/m^2^), ongoing inflammatory diseases [erythrocyte sedimentation rate (ESR) > 30 mm/h, and high-sensitivity C-reactive protein (hs-CRP) > 6 mg/L] and other intercurrent illnesses, poor glycemic control (HbA1c > 8%), smoking, insulin therapy and corticosteroid treatment were exclusion criteria.

All patients underwent anthropometric determinations and blood collection (after a 12 h overnight fasting) for the evaluation of fasting glycemia, HbA1c, lipid profile (total and HDL-cholesterol, and triglycerides), creatinine, and the inflammatory markers ESR and hs-CRP. Microalbuminuria was evaluated according to ADA recommendations (33).

### Pre-analytical Sample Processing

For miRNA studies, approximately 10 mL of venous blood were collected from each patient after 10-12 h fasting in a serum separator tube (Vacutainer® SST™) and centrifuged within 1 h at 2,000 x g for 15 min at 4°C. Serum fractions were rapidly aliquoted into cryovial tubes and stored at −80°C for subsequent use. Prior to further processing, serum aliquots were thawed on ice, and subjected to a second centrifugation at 16,000 × g for 5 min at 4°C to discard remaining insoluble material. At this stage, samples were checked spectrophotometrically at 414 nm to exclude hemolysis.

### Circulating miRNA Profiling

Serum-circulating miRNAs were extracted from all patients enrolled in the study as enriched low molecular-weight RNA (LMW RNA), using a commercial column-based system (miRNeasy Serum/Plasma Kit, Qiagen). Briefly, for miRNA extraction, 200 μL of serum samples were used, and procedures were those recommended by the manufacturer, including the addition of synthetic exogenous *C. elegans* miR-39 for the evaluation of both miRNA extraction efficiency and data normalization. After miRNA quantification and purity control (A_260/280_ ratio = 1.9–2.1 and A_260/230_ ratio = 2.0–2.2) with a spectrometric method (Nanodrop 1000, Thermo Scientific), yielding between 14 and 30 ng RNA/μL (elution volume = 12 μL), equal amounts (100 ng) of RNA extracted from serum samples of well-selected, matched patients with type 2 diabetes, either with (*n* = 5) or without (*n* = 5) DR, were pooled and run at the same time. The LMW RNA fraction (a total of 500 ng for each RNA pool) was used as starting material for a subsequent one-step universal tailing reverse transcription reaction, carried out in a 100 μL reaction volume, using miScript II RT Kit (Qiagen) and a thermocycler system (GeneAmp 2700 Thermal Cycler, Applied Biosystems). MiRNA profiling of the two pools was performed by RT-qPCR on a 384 well-array, containing spotted forward primers for 372 miRNAs with detectable expression in serum (miScript miRNA PCR Array Human Serum & Plasma 384HC, Qiagen; Supplementary Table 1) and SYBR Green chemistry (miScript SYBR Green PCR Kit, Qiagen). Spike-in *C. elegans* miR-39, and 6 reference controls (SNORD61, SNORD68, SNORD72, SNORD95, SNORD96A, and RNU6B/RNU6-2) were used as normalizers. Reactions were carried out on QuantStudio™ 12K Flex Real-Time PCR System (Applied Biosystems), as indicated by the manufacturer's protocol. A free online data analysis software (https://geneglobe.qiagen.com/it/) was used for quality assessment and miRNA expression analysis. Fold-change values >2 and <0.5 were considered significant. MiRNAs with CT values > 30 were excluded.

### Individual Validation

LMW RNA was extracted from serum as described above. After reverse transcription reaction using 50 ng RNA as a template (miScript II RT Kit, Qiagen), individual validation of selected miRNAs was performed using RT-qPCR in all enrolled individuals (miScript SYBR Green PCR Kit, Qiagen; QuantStudio™ 12K Flex Real-Time PCR System, Applied Biosystems). Protocols were those indicated by the manufacturer. Normalization was obtained using the spike-in control *C. elegans* miR-39. All samples were tested in triplicates. A free online data analysis software (https://geneglobe.qiagen.com/it/) was used for quality assessment and miRNA expression analysis.

### Cell Culture

Human retinal pigment epithelial cells (ARPE-19) were cultured in Dulbecco's Modified Eagle Medium (DMEM)/F12 (Sigma Aldrich) supplemented with 10% fetal bovine serum (FBS) and 2 mM L-glutamine; human umbilical vein endothelial cells (HUVECs) were grown in ECM medium (ScienCell Research Laboratories), with the addition of 5% FBS. Penicillin (100 U/mL) and streptomycin (100 μg/mL) were added to each culture medium. All cultured cells were kept at 37°C in a humidified incubator with 5% CO_2_ atmosphere. For glucose treatment experiments, ARPE-19 and HUVEC cells were exposed to 5 or 25 mM glucose, and VEGFA expression was evaluated after 48 h incubation. Mycoplasma contamination was routinely evaluated, and only mycoplasma-free cells were used.

### RNA Extraction and RT-qPCR From Cultured Cells

RNA extraction from ARPE-19 and HUVEC cells was performed using the Trizol reagent (Thermo Fisher Scientific) according to the manufacturer's protocol, followed by DNase treatment (Ambion) to eliminate DNA contaminations. cDNAs were obtained from 1 μg of total RNA, using the High-Capacity cDNA Reverse Transcription Kit (Thermo Fisher Scientific). Sequence-specific primers for human *VEGF* and *RPS9* were as already reported (19, 34). An Eppendorf Mastercycler ep realplex ES real-time thermocycler was used to perform RT-qPCR (19). SYBR Green fluorescence was measured and relative quantification was made against the *RPS9* cDNA, used as an internal standard. All PCR reactions were performed in triplicate.

### LMW RNA Extraction From Cell Supernatants and Lysates, and RT-qPCR

Extraction of the LMW RNA fraction containing miRNAs was obtained by a commercial column-based system (RNeasy MinElute Spin Columns, Qiagen), suitable for both cell supernatants and lysates. In our experimental conditions, 800 μL of supernatant or 700 μL of cell lysates (from 2 × 10^6^ cells) were loaded per column. Fifty nanograms of RNA were used as template for reverse transcription using the miScript II RT Kit (Qiagen) and a thermocycler system (GeneAmp 2700 Thermal Cycler, Applied Biosystems). RT-qPCR was performed using miScript SYBR Green PCR Kit (Qiagen) and Eppendorf Mastercycler ep realplex ES real-time thermocycler (Eppendorf). Normalization was obtained using the spike-in control *C. elegans* miR-39.

### Protein Extraction and Western Blotting

Total cellular extracts were obtained as previously described (35), and Western blot analyses were performed to determine VEGFA protein expression in both ARPE-19 and HUVEC cells, using an anti-VEGFA specific antibody (sc-507, Santa Cruz Biotech).

### Plasmid and miRNA Mimic Transient Transfection

The human VEGFA promoter construct, pGL3-2.6 (a kind gift from Dr. Sheehy, University College Dublin, Ireland) was transiently transfected into HUVEC cells, using LipofectAMINE 2000 reagent (Invitrogen Life Technology), and luciferase activity was assessed 48 h later using the dual-luciferase reporter assay system (Promega). Transient transfection of custom-synthesized *miR-1281* mimic (miScript miRNA Mimics, Qiagen) was carried out using 5 nM mimic in the presence of HiPerFect Transfection Reagent (Qiagen), according to the manufacturer's protocol. *VEGF* gene expression was assessed 48 h post-transfection.

### Wound Healing

HUVEC and ARPE-19 cells were seeded in 12-well plates (0.2 × 10^6^ cells/well, and 0.1 × 10^6^ cells/well, respectively) to obtain confluency within 24 h, transfected with 150 ng (5 nM) miR-1281 mimic, and maintained in high glucose (25 mM) medium for 48 h, after which a wound was mechanically produced by scratching cell monolayers with a sterile 200-μL pipette tip. Cell migration was evaluated by quantitative image analysis of wound closure at 0, 4, 8, and 12 h for HUVEC cells, and up to 30 h for ARPE-19 cells after injury, using the ImageJ software (36). Percent of wound closure was calculated using the following equation:

$$\begin{array}{l} \%\;Wound\;Closure=\frac{Area_{T0}-Area_{Ti}}{Area_{T0}}x\;100 \end{array}$$

### Statistical Analysis

Initially, each quantitative trait was tested for normality of distribution, using the Shapiro-Wilk normality test and, when required, it was log-transformed. Data were expressed as mean ± standard deviation (SD). The non-parametric Mann-Whitney U-test was used to compare intergroup differences in serum miRNA expression and other biochemical parameters. Spearman rank correlation analysis was used to explore the correlation between semiquantitative concentrations of screened miRNAs and clinical and biochemical parameters. Selected miRNAs were then forced in multivariable regression model adjusted for appropriate covariates. Receiver operating characteristic (ROC) analysis was performed to assess the discriminative capacity of selected miRNAs in identifying DR. A *p* < 0.05 was considered statistically significant. Data were analyzed using SPSS software version 20 (SPSS Inc.) and the miScript miRNA PCR array data analysis software (https://geneglobe.qiagen.com/it/).

## Results

### Circulating miRNA Profiling and Individual miRNA Validation

**Figure 1:**
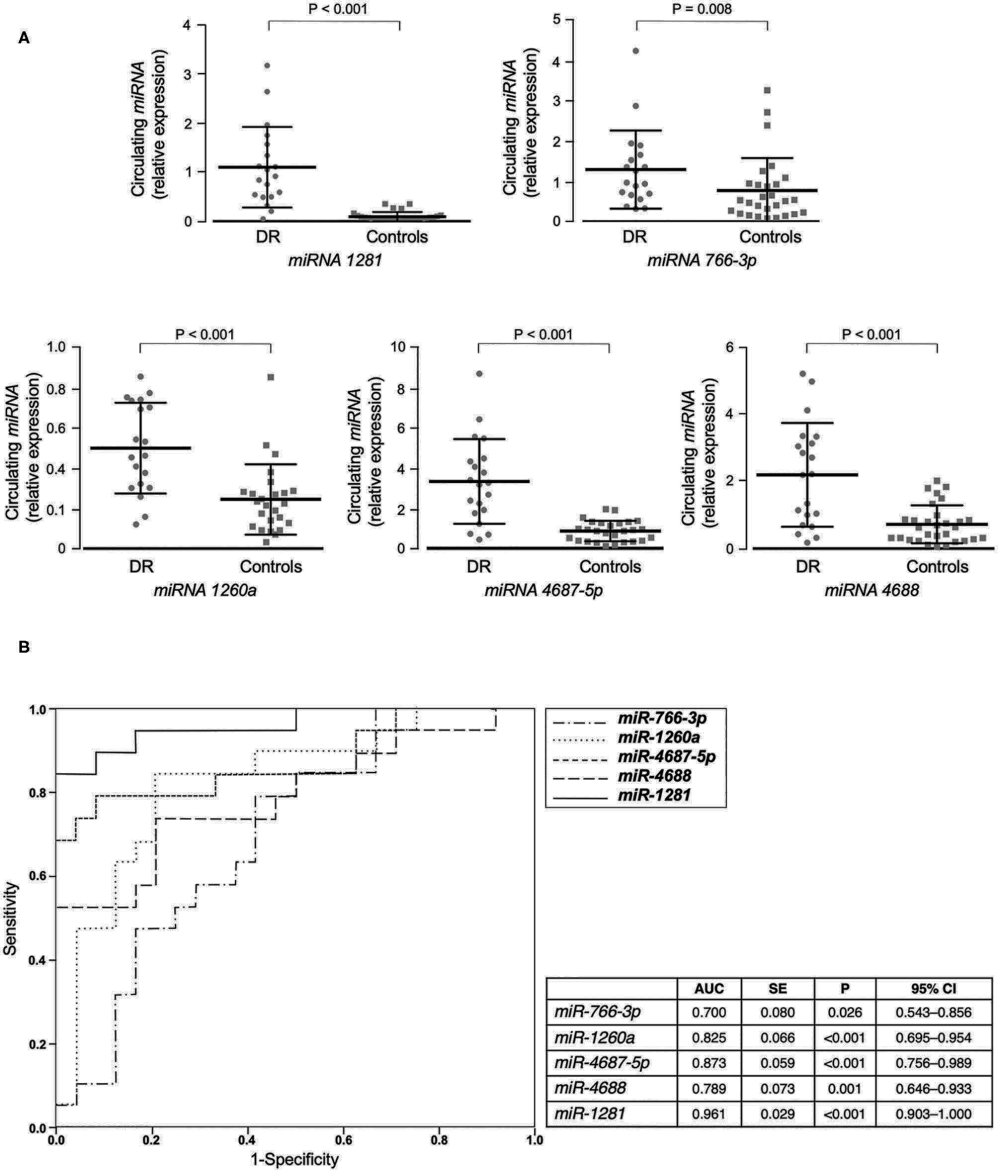
miRNAs validation and miRNA's accuracy. **(A)** Mean ± SD of validated circulating miRNAs in the serum of patients with type 2 diabetes, with (DR, *n* = 20) and without (controls, *n* = 25) DR. Expression levels of selected miRNAs were analyzed by RT-qPCR. The relative quantification measures the concentration of a specific miRNA and is calculated as 2^(−Δ*C*_t_) for each sample (37). ΔC_t_ values are calculated as the raw C_t_ value—average of raw C_t_ values for the selected reference miRNA (cel-miR-39-3p) in each sample. Non-parametric Mann-Whitney test was used for comparisons among two groups. **(B)** ROC curves of validated miRNAs for the prediction of DR. Both sensitivity and specificity are indicated. AUC, area under curve; SE, standard error; CI, confidence interval.

We first performed miRNA profiling on pooled sera from a subgroup of 5 well-selected male patients with type 2 diabetes and non-proliferative DR (cases), and 5 matched patients with type 2 diabetes, but without DR (controls). Among the 372 miRNAs analyzed, 40 were up-regulated and 3 were down-regulated in pooled cases vs. pooled controls, following the threshold of ≤30 CT value and at least 2 average fold change (Table 2). In an attempt to perform a more stringent selection of differentially expressed miRNAs, only those with at least a 5-fold change in expression were chosen for the next step. Therefore, 23 potential miRNA candidates for DR were analyzed by RT-qPCR in an individual validation analysis, in which 15 further new cases and 25 new age- and BMI-matched controls were added to those analyzed in the first step of the study. As shown in Figure 1A, 5 of the 23 selected miRNAs (*miR-1281, miR-4687-5p, miR-4688, miR-1260a*, and *miR-766-3p*) showed a significant up-regulation in patients with DR compared to control patients. In accordance with the data retrieved from array-profiling, *miR-1281* showed the largest fold change (over 9 average) (Figure 1A). On Spearman univariate correlation analysis, *miR-4687-5p* and *miR-766-3p* were correlated with female gender (ρ = 0.344, *p* = 0.018; ρ = 0.376, *p* = 0.009, respectively) and BMI (ρ = 0.400, *p* = 0.005; ρ = 0.322, *p* = 0.027, respectively), whereas *miR-4688* and *miR-1260a* were correlated with BMI only (ρ = 0.426, *p* = 0.002; ρ = 0.298, *p* = 0.049, respectively). Interestingly, *miR-1281* expression was significantly correlated solely with DR (ρ = 0.783), *p* < 0.001), thereby suggesting specificity for this diabetic complication. The association of *miR-1281* with DR was further corroborated by multiple linear regression analysis (beta = 0.689, *t* = 6.632, *p* < 0.001), in which age, sex, BMI, and duration of diabetes were added as covariates.

**Table 2:** Differential miRNAs expression in retinopatic patients, vs. diabetic controls.

**Mature ID**		**Fold regulation**	**Ct DR**	**Ct control**
1	hsa-miR-1281	13.88	27.26	27
2	hsa-miR-328-3p	13.26	29.78	27.86
3	hsa-miR-4688	12.17	29.08	28.43
4	hsa-miR-1260a	10.02	27.63	26.9
5	hsa-miR-4687-5p	9.25	27.27	25.86
6	hsa-miR-19a-3p	8.85	29.53	26.91
7	hsa-miR-490-3p	8.59	29.78	27.32
8	hsa-miR-766-3p	8.56	28.16	29.13
9	hsa-miR-3135b	8.18	28.31	28.44
10	hsa-miR-574-3p	7.85	28.71	26.75
11	hsa-miR-637	7.74	28.83	28.38
12	hsa-miR-532-3p	7.69	29.6	29.69
13	hsa-miR-197-3p	7.59	29.18	27.21
14	hsa-miR-4286	6.55	28.35	25.73
15	hsa-miR-489-3p	6.3	28.83	28.14
16	hsa-miR-628-3p	6.27	28.99	28.43
17	hsa-miR-4301	6.25	28.55	27.14
18	hsa-miR-3907	6.17	29.77	28.34
19	hsa-miR-7-2-3p	6.04	28.77	28.18
20	hsa-miR-150-5p	5.88	27.41	25.91
21	hsa-miR-195-5p	5.8	27.9	26.38
22	hsa-miR-4538	5.3	26.02	22.36
23	hsa-miR-16-5p	5.01	27.44	27.15
24	hsa-miR-342-3p	4.39	28.25	24.11
25	hsa-miR-720	4.27	29.95	29.15
26	hsa-miR-126-5p	4.24	29.18	27.21
27	hsa-miR-30a-5p	3.99	29.1	28.98
28	hsa-miR-324-3p	3.8	29.58	27.45
29	hsa-miR-21-5p	3.57	28.74	26.52
30	hsa-miR-223-3p	3.06	28.73	25.95
31	hsa-miR-126-3p	3.02	25.79	23.35
32	hsa-miR-423-5p	2.9	29.89	27.37
33	hsa-miR-1280	2.76	26.91	24.32
34	hsa-miR-23a-3p	2.7	26.97	25.24
35	hsa-miR-320a	2.7	29.57	27.51
36	hsa-miR-25-3p	2.42	29.87	28.96
37	hsa-miR-4454	2.34	27.42	24.59
38	hsa-miR-451a	2.21	24.58	21.67
39	hsa-miR-484	1.85	29.59	26.42
40	hsa-miR-4516	1.25	29.25	25.52
41	hsa-miR-373-5p	−1.06	29.36	29.94
42	hsa-miR-486-5p	−1.31	26.64	23.2
43	hsa-miR-92a-3p	−1.53	27.6	26.97

Furthermore, ROC curve analyses were performed to assess sensitivity and specificity of selected circulating miRNAs in detecting DR. As shown in Figure 1B, all five selected miRNAs showed a significant area under the curve (AUC) and, among them, *miR-1281* stood out as the best predictor of DR [AUC = 0.965 (0.902–1.000), *p* < 0.001], thus suggesting this miRNA as a potential novel biomarker for early diagnosis of DR.

### Effect of Glucose Concentration on *miR-1281* and VEGFA Expression

**Figure 2:**
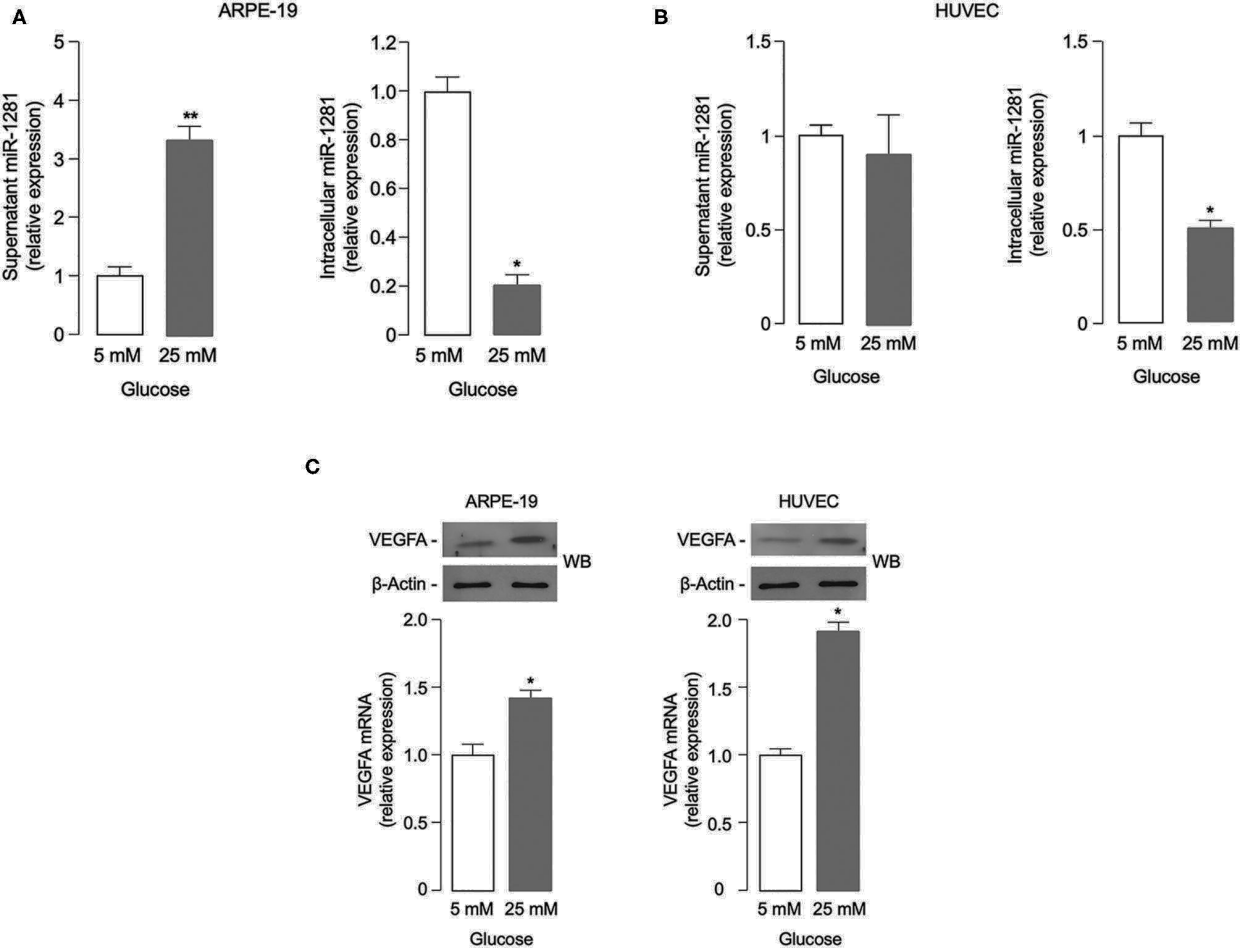
Effect of glucose on *miR-1281* and VEGFA expression. **(A,B)** ARPE-19 epithelial cells and HUVEC endothelial cells were exposed to low (5 mM) or high (25 mM) glucose, and both extracellular (white bar) and intracellular (gray bar) levels of *miR-1281* were obtained after 48 h incubation. Results are the mean ± SEM of three independent experiments, each in triplicate. *p < 0.05; **p < 0.001. **(C)** VEGFA mRNA and protein expression in ARPE-19 and HUVEC cells, as measured by RT-qPCR (bar graphs) and western blot (WB), respectively. mRNA levels are shown as means ± SEM of three independent experiments, each performed in triplicate. *p < 0.05. Representative WBs of VEGFA protein in both cell types are representative of at least 3 independent experiments. β-Actin, control of protein loading.

To investigate the potential role of *miR-1281* in retinal damage, we first evaluated whether *miR-1281* could be affected by glucose levels *in vitro*, in both ARPE-19 retinal epithelial cells and HUVEC endothelial cells exposed for 48 h to high (25 mM) glucose, a condition simulating that observed during sustained hyperglycemia. As shown in Figure 2A, *miR-1281* was increased in the supernatant medium of cultured ARPE-19 cells, and decreased in the intracellular fraction in response to high glucose (*p* < 0.001), as measured by RT-qPCR. Under the same experimental conditions as those used for ARPE-19 cells, no differences in *miR-1281* levels were observed in the extracellular fraction of HUVEC cells in relation to media glucose concentration, whereas *miR-1281* was down-regulated in the intracellular location of HUVEC cells treated with high glucose (Figure 2B). In both cell lines, exposure to high glucose resulted in the up-regulation of VEGFA mRNA and protein expression compared to cells exposed to low (5 mM) glucose (*p* < 0.05 for ARPE cells; *p* < 0.01 for HUVEC cells) (Figure 2C), suggesting that a link exists between glucose levels, the release of *miR-1281*, and the expression of VEGFA.

### Functional Significance of *miR-1281* in *VEGFA* Gene Expression

**Figure 3:**
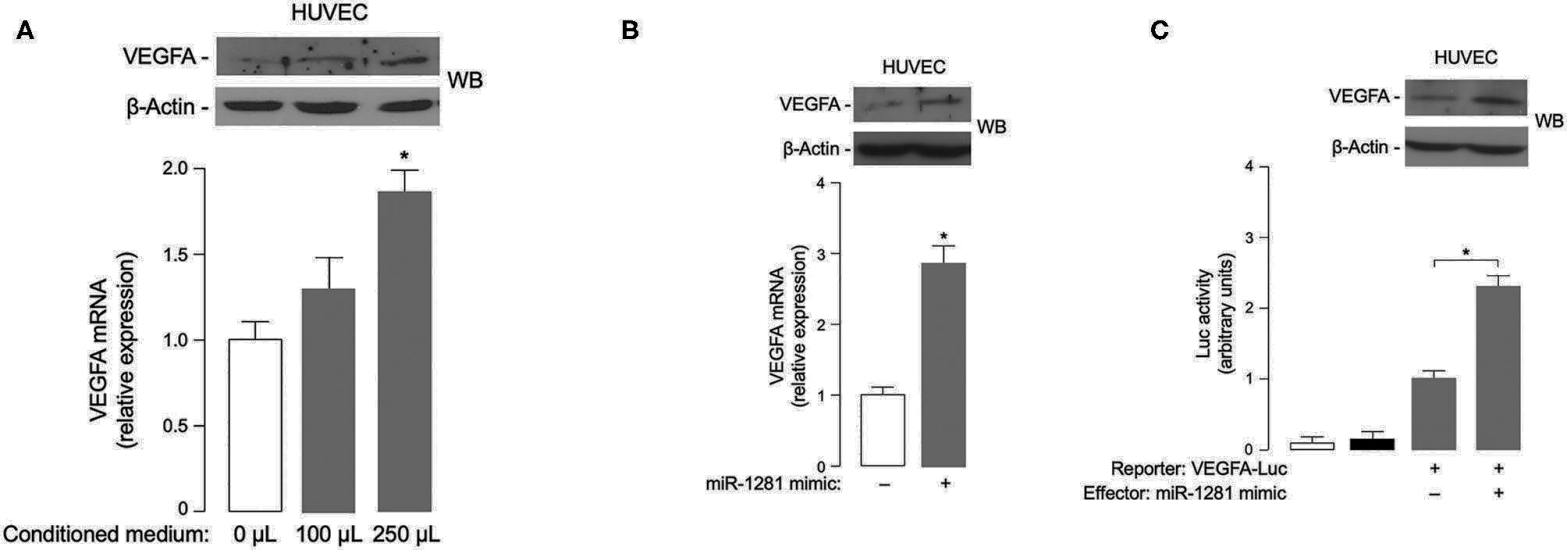
Effects of *miR-1281* on VEGFA expression and cell migration. **(A)** VEGFA expression in HUVEC cells exposed to increasing amounts (0, 100, 250 μL) of conditioned medium from ARPE-19 cells, which underwent high glucose-induced *miR-1281*. VEGFA mRNA and protein levels were measured 48 h later by RT-qPCR and WB, respectively. VEGFA mRNA data are expressed as means ± SEM of three independent measurements, each performed in triplicate. *p < 0.05 vs. control (white bar). A representative WB of VEGFA out of three independent experiments is shown in the autoradiogram. β-actin, control of protein loading. **(B)** VEGFA mRNA and protein levels were measured as in **(A)**, in HUVEC cells untreated or pretreated with 150 ng *miR-1281 mimic* for 48 h. Data for RT-qPCR and WB are representative of at least 3 independent experiments. *p < 0.05 vs. control (white bar). **(C)** Human VEGFA-Luc reporter vector (2 μg) was transfected into HUVEC cells, in the absence or presence of *miR-1281 mimic* (150 ng), and Luc activity was measured 48 h later. Data are means ± SEM for three separate transfections. Values are expressed as the factor of increase above the level of Luc activity obtained in cells transfected with VEGFA-Luc reporter vector in the absence of *miR-1281 mimic* (control), which is assigned an arbitrary value of 1. White bar, mock (no DNA); black bar, pGL3-basic (effector vector without an insert). *p < 0.05 vs. control. A representative WB of endogenous VEGFA in untreated and *miR-1281 mimic*-treated cells is shown in the autoradiogram.

Based on the involvement of VEGFA in DR (4), it was intriguing to hypothesize that *miR-1281* could be related to the microangiopathic damage of retinal vessels by up-regulating the expression of VEGFA, whose intimate role in retinal neovascularization and retinal hemorrhage is well-established (21). In an attempt to test this hypothesis, we examined the effect of *miR-1281-*enriched medium from *in vitro* cell cultures on VEGFA expression. As shown in Figure 3A, treatment with increasing amounts of conditioned medium from ARPE-19 epithelial cells exposed to high glucose caused a rise in both *VEGFA* mRNA and protein expression in HUVEC endothelial cells, thereby consistently supporting the hypothesis that the adverse effect of high glucose on retinal endothelial cell injury can be mediated, at least in part, by *miR-1281* via VEGFA.

To explore the possibility that *miR-1281* independently directed *VEGFA* gene expression, transient transfection studies were undertaken using *miR-mimic 1281* in HUVEC cells. As shown in Figure 3B, *VEGFA* mRNA expression was considerably increased in miR-mimic treated HUVEC cells as compared to miR-mimic negative control transfected cells. The increase in mRNA abundance paralleled the increase in VEGFA protein expression as detected by Western blot analysis of nuclear extracts from HUVEC cells transfected with *miR-mimic 1281* (Figure 3B). Next, to test whether the *VEGFA* gene promoter could be transcriptionally regulated by *miR-1281*, HUVEC cells were transiently co-transfected with the luciferase (Luc) reporter plasmid *VEGF 2.6-Luc*, together with *miR-1281* mimic. Under these experimental treatments, *miR-1281* induced a significant increase in *VEGF-Luc* activity, which was paralleled by an increase in VEGFA protein abundance (Figure 3C), thereby indicating that *miR-1281* positively regulates VEGFA protein expression through activation of *VEGFA* gene transcription.

### Role of *miR-1281* on Cell Migration

**Figure 4:**
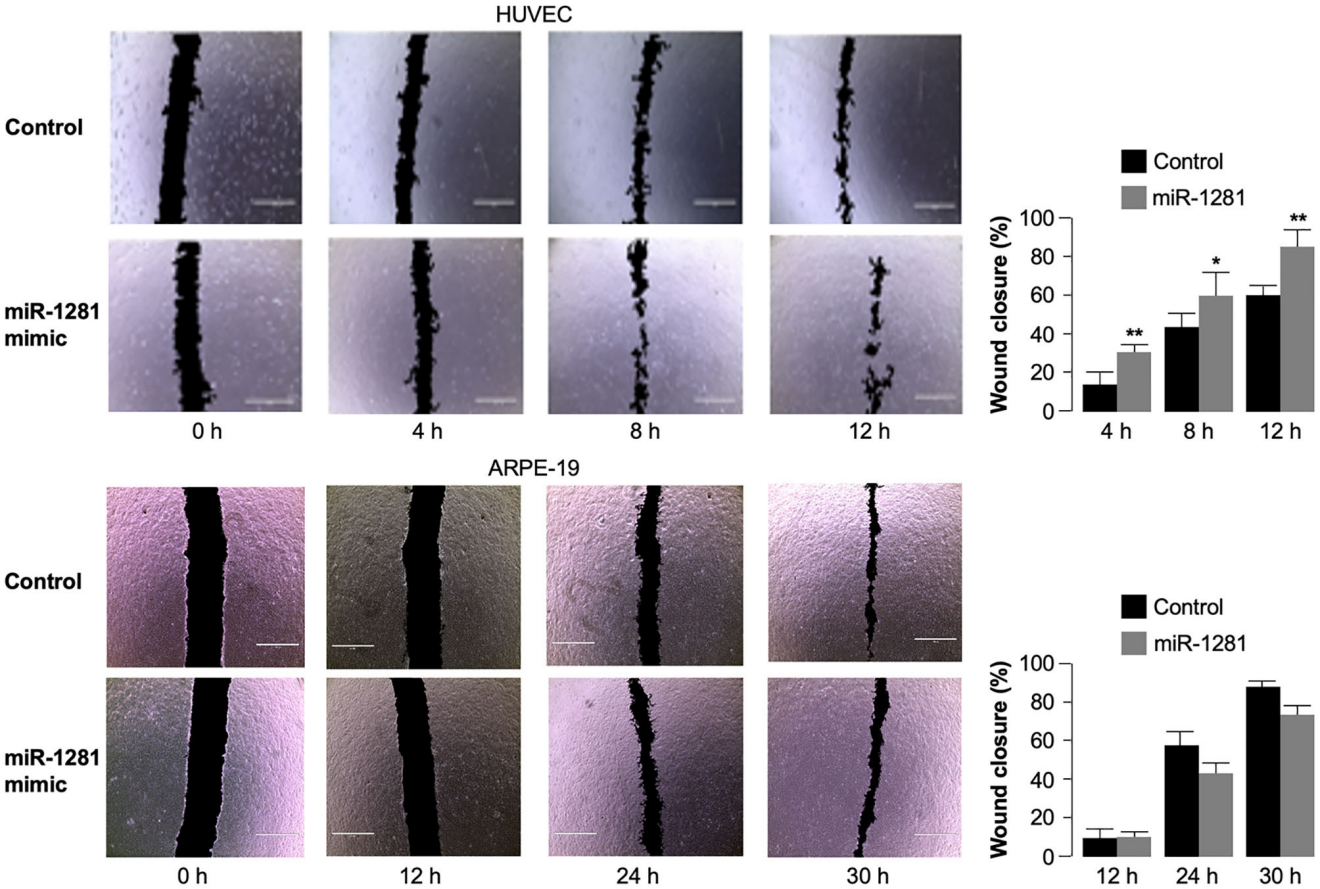
Effect of *miR-1281* on cell migration. Wound healing assays were performed in HUVEC and ARPE-19 cells, either untransfected (Control), or transfected with *miR-1281 mimic* for 48 h. Wound closure was evaluated at the indicated time, after cell scratch, by phase contrast imaging (total magnification: 40 x), and analyzed with the ImageJ software. Quantitative data are expressed in bar graphs as mean ± SD of three independent assays, in which at least three images per condition were obtained. *p < 0.05; **p < 0.01 relative to untreated control cells at each time point.

To investigate the biological role of *miR-1281* in relation to the molecular effects described on VEGFA expression, we finally performed wound healing assays in HUVEC cells to study cell migration and cell interactions *in vitro*. After cell transfection with *miR-1281* mimic, we analyzed the closure of wounds mechanically created on the HUVEC cell monolayer as a surrogate of cell migration during wound healing *in vivo*. The images from cell scratch assay showed that directional cell migration was increased in *miR-1281* transfected cells, in which wound closure was significantly enhanced compared with control cells at either 4, 8, or 12 h after scratch (Figure 4). In contrast, no differences in cell migration were found in ARPE-19 cells transfected or untransfected with *miR-1281* mimic, in which wound closure following scratch occurred considerably later with respect to HUVEC cells (Figure 4). As proliferation and migration of retinal endothelial cells have been associated with the development of DR, these results sustain a role of *miR-1281* in the regulation of factors known to be involved in this process, which include, among others, the VEGFA.

## Discussion

In the present study, we investigated the differential expression of circulating miRNAs in a group of patients with type 2 diabetes, with and without non-proliferative DR. After comprehensive serum miRNA profiling and validation of individual miRNAs, 5 circulating miRNAs appeared to be highly up-regulated in patients with DR, compared to diabetic patients without DR. In particular, one of them, *miR-1281*, was the most up-regulated and seemed to be more specifically related to DR, displaying the strongest sensitivity and specificity in detecting this microvascular complication of diabetes.

In recent years, several works have investigated the association of miRNAs with DR (23–26, 37–52). In circulating miRNA profiling studies, miR-27b, miR-320a, and miR-126 were validated in patients with type 1 diabetes and DR (23, 42). In similar studies, miR-21, miR-181c, and miR-1179 were significantly upregulated in type 2 diabetic patients with proliferative DR vs. patients with non-proliferative DR (25), while miR-3197 and miR-2116-5p were higher in diabetic patients with DR, compared with patients without DR (37). In addtion, using an RNA-Seq approach, it was evidenced that miR-4448, miR-338-3p, miR-190a-5p, miR-485-5p, and miR-9-5p appeared differentially expressed in serum from retinopathic diabetic patients when compared to controls (49). However, while validation of miR-21 and miR-126 in DR was confirmed in other similar studies (39, 40, 44, 45), miR-320a, which was previously found to be upregulated in DR (23), was later reported to be significantly downregulated in another work (51), indicating, therefore, that discrepancies exist in this context. Several explanations can be provided for the lack of consensus in the literature in this regard. First, the selection of patients is very critical, as ethnicity, age, inclusion and exclusion criteria may substantially differ among studies. Second, differences in pre-analytical and analytical procedures may also account for discrepancies in the results. Third, there is no agreement on how miRNAs should be normalized, thus representing another issue in this debate. We believe that despite the remarkable number of studies on DR, all these observations indicate that additional miRNAs, including *miR-1281*, might still emerge as predictive indexes for DR.

Until now, only few studies have been published in which authors had identified *miR-1281* as a potential biomarker in human diseases. Among these disease states were abdominal aortic aneurysm (53), coronary heart disease and pulmonary arterial hypertension (54), chronic nephropathy (55), immune thrombocytopenic purpura (56), and malignant pleural mesothelioma (57). Our findings in the present work are consistent with these studies, implicating *miR-1281* as a potential biomarker to track disease progression or aid in disease diagnosis. Furthermore, our results in the present work are in agreement with other studies indicating that *miR-1281* is up-regulated in the central cornea of patients with diabetes (58), as well as in plasma and urine samples of patients with chronic kidney disease, a condition that is often secondary to poorly controlled diabetes (55).

Apart from the histone deacetylase 4 (*HDAC4*) gene, that has been recently shown as a direct target of *miR-1281* in pulmonary artery smooth muscle cells (54), no other targets of *miR-1281* have been identified yet. Our *in vitro* results from human ARPE-19 retinal cells show that the extracellular release of *miR-1281* following high glucose treatment significantly exceeded the release under low glucose conditions, and this behavior only partially reflected that of endothelial HUVEC cells, in which *miR-1281* was also significantly reduced in the intracellular environment, although to a lesser extent than in ARPE-19 cells, but was unchanged in the extracellular medium. Based on these data, and on the observation that treatment with *miR-1281* resulted in the increase in both VEGFA mRNA abundance and VEGFA promoter-driven Luc activity in HUVEC cells, it can be speculated that increased release of *miR-1281* by epithelial retinal cells in instances of chronic glucose excess, such as in diabetes, might play a pathogenetic role in DR through the activation of VEGFA protein production from adjacent endothelial cells, thereby inducing VEGFA-mediated neoangiogenesis and vascular damage of retinal vessels. How miR-1281 upregulates VEGFA gene transcription is not clear and requires further studies. TargetScan prediction analysis suggests that this miRNA could potentially target mRNA sequences of the hypoxia-inducible factor-1α inhibitor (HIF1AN). The accumulation of HIF-1α and the impairment of Von Hippel-Lindau functional activity, due to miR-1281-induced HIF1AN instability/degradation, would both increase HIF-1—a master regulator of hypoxia—and, as a consequence, the enhanced transcription of hypoxia-responsive genes, including VEGFA. Whether miR-1281 has a functional role in glucose-induced retinal damage by VEGFA via HIF-1 has to be proved. If this interpretation is correct, it seems tempting to imply *miR-1281* as a novel pathogenetic marker of retinal microangiopathic damage in patients with diabetes, which may explain, at least in part, how chronic sustained hyperglycemia could abnormally up-regulate VEGFA in retinal cells. Furthermore, the stimulatory effect of *miR-1281* on HUVEC cell migration observed in this study indeed supports the biological role of this miRNA in DR. In this context, VEGFA definitely plays a role, although the potential link of *miR-1281* to other factors involved in the directional migration of retinal endothelial cells, such as adhesion molecules (59), cannot be excluded.

On the other hand, the involvement of selected miRNAs in a growing number of pathophysiological conditions, including diabetes and its microvascular complications, has also been reported by others, in both clinical and non-clinical settings (14, 17). As reported above, the attempt to identify a sensitive and specific biomarker related to DR among circulating miRNAs has produced, so far, poor consistency between results (21, 60–62). Although other miRNAs may also contribute to the development of DR (17, 63–65), our results herein indicate, for the first time, that *miR-1281* could indeed be a potential novel biomarker of DR. Compared to other studies, the main strengths of our work are the enrollment of highly selected patients with type 2 diabetes and their genetic homogeneous makeup with no genetic admixture (66). Also, of particular interest is the simultaneous demonstration as to how overproduction of *miR-1281* in retinal cells may pathophysiologically contribute to retinal vascular damage, providing a mechanistic explanation for the adverse effect of this miRNA on DR. This should contribute to exceed the main limit of our study, mainly represented by its small sample size, in addition to differences in triglyceride levels in DR patients vs. controls as possible confounder to the results of this work.

## Conclusion

Our findings identify *miR-1281* as a novel sensitive and specific non-invasive biomarker for the early detection of DR. Furthermore, they suggest that, by positively regulating the expression of the neoangiogenic factor VEGFA in HUVEC endothelial cells under glucose excess conditions, *miR-1281* can play a pathogenic role in the development of DR. Further studies with larger numbers of patients are required in order to fully validate these results in a clinical setting of patients with diabetes.

## Data Availability Statement

The raw data supporting the conclusions of this article will be made available by the authors, without undue reservation.

## Ethics Statement

The studies involving human participants were reviewed and approved by Comitato etico regionale, Sezione Area Centro, Catanzaro, Italy, Protocol no. 116, May 14, 2015. The patients/participants provided their written informed consent to participate in this study.

## Author Contributions

MG, FA, DC, and BA performed experiments *in vitro* and validation of selected miRNAs in individual samples. MG and EC analyzed data, performed statistical analysis and contributed to manuscript draft. MM and RL helped collecting blood samples and clinical data from ambulatory patients. FB provided valuable suggestions and drafted figures. SP and AF contributed reagents, materials. VS performed ophthalmological examinations for diabetic retinopathy. DF conceived and helped supervise the project and contributed to data interpretation. AB conceived and supervised the study and wrote the manuscript. All authors contributed to the article and approved the submitted version.

## Conflict of Interest

The authors declare that the research was conducted in the absence of any commercial or financial relationships that could be construed as a potential conflict of interest.
