# Supplementary material for: MicroRNA-1281 as a Novel Circulating Biomarker in Patients With Diabetic Retinopathy
Source: Front Endocrinol (Lausanne). 2020 Aug 4;11:528. doi: 10.3389/fendo.2020.00528 (PMC7417427; doi:10.3389/fendo.2020.00528)
Supplement: Supplementary file 1 [file Table_1.DOCX]

Supplementary Material

**Supplementary Table**. miRNAs forward primers in 384HC Array

| **Mature miRNA ID** | | | | | |
| --- | --- | --- | --- | --- | --- |
| hsa-miR-495-3p | hsa-miR-1180-3p | hsa-miR-125b-5p | hsa-miR-98-5p | hsa-miR-4302 | hsa-miR-625-3p |
| hsa-let-7c-5p | hsa-miR-133b | hsa-miR-203a-3p | hsa-miR-144-3p | hsa-miR-183-5p | hsa-miR-15b-3p |
| hsa-miR-3141 | hsa-miR-324-3p | hsa-let-7i-5p | hsa-miR-20a-5p | hsa-miR-27b-3p | hsa-miR-30a-5p |
| hsa-miR-184 | hsa-miR-3135b | hsa-miR-454-3p | hsa-miR-195-5p | hsa-miR-15b-5p | hsa-miR-206 |
| hsa-miR-532-5p | hsa-miR-502-3p | hsa-miR-3159 | hsa-miR-3191-3p | hsa-miR-424-5p | hsa-miR-4538 |
| hsa-miR-152-3p | hsa-miR-3655 | hsa-let-7f-1-3p | hsa-miR-4454 | hsa-miR-373-5p | hsa-miR-1237-3p |
| hsa-miR-1231 | hsa-miR-130b-5p | hsa-miR-548o-5p | hsa-miR-766-3p | hsa-miR-374c-5p | hsa-miR-19b-3p |
| hsa-miR-3911 | hsa-miR-324-5p | hsa-miR-1913 | hsa-miR-99b-5p | hsa-miR-375 | hsa-miR-142-3p |
| hsa-miR-223-5p | hsa-miR-373-3p | hsa-miR-548e-3p | hsa-miR-140-5p | hsa-miR-574-3p | hsa-miR-30e-5p |
| hsa-miR-1909-5p | hsa-miR-3646 | hsa-miR-4770 | hsa-let-7d-3p | hsa-miR-4524a-3p | hsa-miR-499a-5p |
| hsa-miR-4291 | hsa-miR-1-3p | hsa-miR-186-5p | hsa-miR-328-3p | hsa-miR-191-3p | hsa-miR-1307-3p |
| hsa-miR-205-5p | hsa-miR-3907 | hsa-miR-10a-5p | hsa-miR-652-3p | hsa-miR-17-5p | hsa-miR-500a-5p |
| hsa-miR-3651 | hsa-miR-422a | hsa-miR-1203 | hsa-miR-133a-3p | hsa-miR-7-1-3p | hsa-miR-204-5p |
| hsa-miR-1587 | hsa-miR-338-5p | hsa-miR-1280 | hsa-miR-379-5p | hsa-miR-720 | hsa-miR-20b-5p |
| hsa-miR-372-3p | hsa-miR-631 | hsa-miR-101-3p | hsa-miR-27a-3p | hsa-miR-675-3p | hsa-miR-200c-3p |
| hsa-miR-1260a | hsa-miR-143-3p | hsa-miR-1910-5p | hsa-miR-664a-3p | hsa-miR-128-3p | hsa-miR-190a-5p |
| hsa-miR-19b-1-5p | hsa-miR-627-5p | hsa-miR-151a-3p | hsa-miR-185-5p | hsa-miR-23b-5p | hsa-miR-193b-3p |
| hsa-let-7a-3p | hsa-let-7i-3p | hsa-miR-196a-5p | hsa-miR-523-5p | hsa-miR-181c-3p | hsa-miR-425-3p |
| hsa-miR-183-3p | hsa-miR-451a | hsa-miR-877-5p | hsa-miR-4258 | hsa-let-7f-5p | hsa-miR-576-5p |
| hsa-miR-1290 | hsa-miR-29b-3p | hsa-miR-148b-3p | hsa-miR-141-3p | hsa-miR-1225-3p | hsa-miR-590-3p |
| hsa-miR-628-3p | hsa-miR-194-5p | hsa-miR-100-5p | hsa-miR-370-3p | hsa-miR-301b-3p | hsa-miR-10b-5p |
| hsa-miR-92b-3p | hsa-miR-605-5p | hsa-miR-4422 | hsa-let-7f-2-3p | hsa-miR-26b-5p | hsa-miR-96-5p |
| hsa-miR-7-2-3p | hsa-miR-30b-5p | hsa-miR-18a-3p | hsa-miR-23b-3p | hsa-miR-885-5p | hsa-miR-1277-3p |
| hsa-miR-144-5p | hsa-miR-199a-5p | hsa-miR-15a-5p | hsa-miR-378h | hsa-miR-4286 | hsa-miR-486-5p |
| hsa-miR-3610 | hsa-miR-125a-5p | hsa-miR-134-5p | hsa-miR-122-5p | hsa-miR-3622a-5p | hsa-miR-188-5p |
| hsa-miR-199b-5p | hsa-miR-376c-3p | hsa-miR-421 | hsa-miR-424-3p | hsa-miR-3185 | hsa-miR-615-5p |
| hsa-miR-1286 | hsa-miR-503-5p | hsa-miR-181c-5p | hsa-miR-542-5p | hsa-miR-629-5p | hsa-miR-30a-3p |
| hsa-miR-497-5p | hsa-miR-361-3p | hsa-miR-337-5p | hsa-miR-433-3p | hsa-miR-4296 | hsa-miR-378i |
| hsa-miR-18b-5p | hsa-miR-590-5p | hsa-miR-1537-3p | hsa-miR-140-3p | hsa-miR-31-5p | hsa-miR-93-5p |
| hsa-miR-214-3p | hsa-miR-1207-5p | hsa-miR-7-5p | hsa-miR-1976 | hsa-miR-483-5p | hsa-miR-154-5p |
| hsa-miR-34a-5p | hsa-miR-342-5p | hsa-miR-139-5p | hsa-miR-24-3p | hsa-miR-23a-3p | hsa-miR-425-5p |
| hsa-miR-4732-5p | hsa-miR-1193 | hsa-miR-551a | hsa-let-7d-5p | hsa-miR-301a-3p | hsa-miR-362-3p |
| hsa-miR-660-5p | hsa-miR-624-5p | hsa-miR-15a-3p | hsa-miR-215-5p | hsa-miR-181d-5p | hsa-miR-92a-3p |
| hsa-miR-3120-3p | hsa-miR-4689 | hsa-miR-339-3p | hsa-miR-548d-5p | hsa-miR-99a-5p | hsa-miR-222-3p |
| hsa-miR-151b | hsa-miR-139-3p | hsa-miR-340-5p | hsa-miR-130b-3p | hsa-miR-330-3p | hsa-miR-296-5p |
| hsa-miR-423-5p | hsa-miR-4516 | hsa-miR-30d-5p | hsa-miR-1183 | hsa-miR-335-5p | hsa-miR-365b-3p |
| hsa-miR-4687-5p | hsa-miR-1539 | hsa-miR-3923 | hsa-let-7a-5p | hsa-miR-4289 | hsa-miR-551b-3p |
| hsa-miR-93-3p | hsa-miR-377-3p | hsa-let-7e-5p | hsa-miR-4505 | hsa-miR-146b-5p | hsa-miR-2467-3p |
| hsa-miR-2276-3p | hsa-miR-130a-3p | hsa-miR-335-3p | hsa-miR-208a-3p | hsa-miR-744-5p | hsa-miR-2355-3p |
| hsa-miR-33a-5p | hsa-miR-4323 | hsa-miR-345-5p | hsa-miR-211-5p | hsa-miR-2355-5p | hsa-miR-200b-3p |
| hsa-miR-150-5p | hsa-miR-339-5p | hsa-miR-485-5p | hsa-miR-1247-5p | hsa-miR-16-5p | hsa-miR-18a-5p |
| hsa-miR-21-5p | hsa-miR-501-5p | hsa-miR-3183 | hsa-miR-378g | hsa-miR-490-3p | hsa-miR-142-5p |
| hsa-miR-329-3p | hsa-miR-132-3p | hsa-miR-532-3p | hsa-miR-485-3p | hsa-miR-34c-3p | hsa-miR-487a-3p |
| hsa-miR-550a-5p | hsa-miR-29a-3p | hsa-miR-542-3p | hsa-miR-9-5p | hsa-miR-136-5p | hsa-miR-145-5p |
| hsa-miR-378b | hsa-miR-22-5p | hsa-miR-199b-3p | hsa-miR-148a-3p | hsa-miR-106b-5p | hsa-miR-1233-3p |
| hsa-miR-28-5p | hsa-miR-596 | hsa-miR-337-3p | hsa-miR-5095 | hsa-miR-196b-3p | hsa-miR-369-3p |
| hsa-miR-138-1-3p | hsa-miR-191-5p | hsa-miR-4301 | hsa-miR-409-3p | hsa-miR-378a-5p | hsa-miR-637 |
| hsa-miR-193a-5p | hsa-miR-103a-2-5p | hsa-miR-381-3p | hsa-miR-361-5p | hsa-miR-145-3p | hsa-miR-202-3p |
| hsa-miR-26b-3p | hsa-let-7b-5p | hsa-miR-412-3p | hsa-miR-331-3p | hsa-miR-320e | hsa-miR-942-5p |
| hsa-miR-16-2-3p | hsa-miR-103a-3p | hsa-miR-505-3p | hsa-miR-30c-5p | hsa-miR-874-3p | hsa-miR-4688 |
| hsa-miR-146a-5p | hsa-miR-489-3p | hsa-miR-136-3p | hsa-miR-487b-3p | hsa-miR-4274 | hsa-miR-197-3p |
| hsa-miR-3200-3p | hsa-miR-4306 | hsa-miR-363-3p | hsa-miR-32-5p | hsa-miR-200a-3p | hsa-miR-126-5p |
| hsa-miR-21-3p | hsa-miR-378a-3p | hsa-miR-1301-3p | hsa-miR-3176 | hsa-miR-340-3p | cel-miR-39-3p |
| hsa-let-7g-5p | hsa-miR-30e-3p | hsa-let-7g-3p | hsa-miR-192-5p | hsa-miR-1260b | cel-miR-39-3p |
| hsa-miR-338-3p | hsa-miR-107 | hsa-miR-598-3p | hsa-miR-181a-5p | hsa-miR-126-3p | SNORD61 |
| hsa-miR-2110 | hsa-miR-382-5p | hsa-miR-221-3p | hsa-miR-1287-5p | hsa-miR-326 | SNORD68 |
| hsa-miR-3200-5p | hsa-miR-484 | hsa-miR-28-3p | hsa-miR-378e | hsa-miR-223-3p | SNORD72 |
| hsa-miR-196b-5p | hsa-miR-1281 | hsa-miR-629-3p | hsa-miR-320b | hsa-miR-219a-13p | SNORD95 |
| hsa-miR-769-5p | hsa-miR-3653-3p | hsa-miR-4267 | hsa-miR-671-3p | hsa-miR-25-3p | SNORD96A |
| hsa-miR-29c-3p | hsa-miR-423-3p | hsa-miR-3131 | hsa-miR-374a-5p | hsa-miR-210-3p | RNU6-6P |
| hsa-miR-4651 | hsa-miR-224-5p | hsa-miR-124-3p | hsa-miR-450a-5p | hsa-miR-19a-3p | miRTC |
| hsa-miR-127-3p | hsa-miR-877-3p | hsa-miR-155-5p | hsa-miR-3613-3p | hsa-miR-17-3p | miRTC |
| hsa-miR-29c-5p | hsa-miR-4732-3p | hsa-miR-486-3p | hsa-miR-342-3p | hsa-miR-151a-5p | PPC |
| hsa-miR-181b-5p | hsa-miR-22-3p | hsa-miR-26a-5p | hsa-miR-3689e | hsa-miR-320a | PPC |
|  |  |  |  |  |  |

Sequences are available on *miRBase* *(*[*http://www.mirbase.org*](http://www.mirbase.org)*).*

**
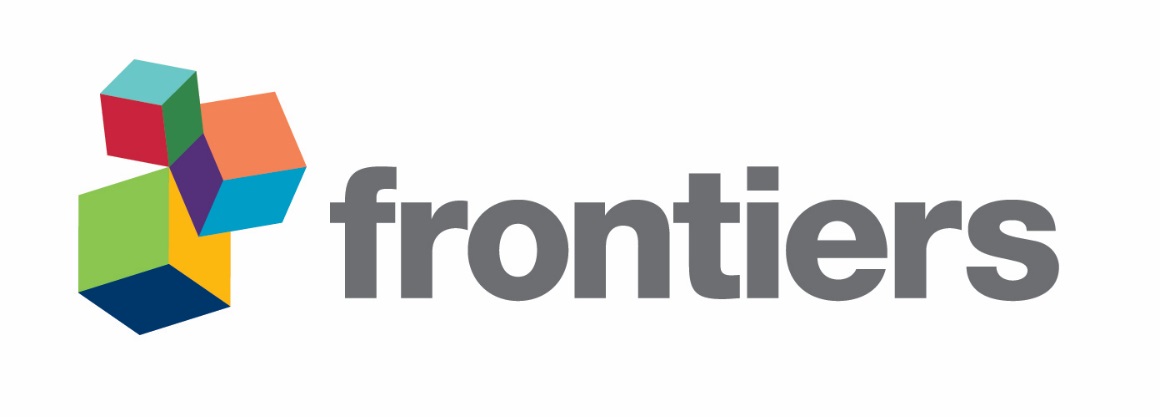
**
